# Dormancy break, sprouting and later tuber reproduction in response to different tuber sizes of tiger nut (*Cyperus esculentus* L.)

**DOI:** 10.1098/rsos.231616

**Published:** 2024-02-14

**Authors:** Binshuo Liu, Hanbo Li, Wei Chen, Ying Wang, Yubo Chen, Xiaowei Wei

**Affiliations:** ^1^ Jilin Jianzhu University, Changchun 130118, People's Republic of China; ^2^ Key Laboratory for Comprehensive Energy Saving of Cold Regions Architecture of Ministry of Education, Jilin Jianzhu University, Changchun 130118, People's Republic of China; ^3^ Institute of Pomology, Jilin Academy of Agricultural Sciences, Gongzhuling 136100, People's Republic of China; ^4^ Jilin Provincial Key Laboratory for Plant Resources Science and Green production, Jilin Normal University, Siping 136000, People's Republic of China

**Keywords:** tuber, dormancy, sprout, later reproduction

## Abstract

Dormancy release pattern, sprout growth and later reproduction were studied among various tuber sizes of *Cyperus esculentus* to determine effective methods to release dormancy and further to select suitable tuber size of this species in production. The results showed that medium tubers performed better during sprouting than large and small tubers under all pre-sprouting treatments. Pre-sprouting treatments at 25°C, 35°C, RT (room temperature) and −2°C were effective in relieving dormancy in medium tubers. Tiller number from medium tubers were significantly higher under 25°C, RT and 45°C than under 35°C and −2°C. Shoot and root mass from medium tubers were significantly higher under the 25°C, 35°C and RT than under other treatments. Tiller and tuber numbers both decreased with decreasing tuber size, as did tuber yield after three months of growth. Furthermore, leftover mass decreased with decreasing tuber mass and remained unchanged at sprouting and maturity periods. A significantly negative allometric correlation was found between plant mass and tuber mass from small tubers. However, a significantly positive allometric correlation was found between tuber size and tuber number from large tubers. In conclusion, medium tubers had a competitive advantage in sprouting, growth and reproduction.

## 1. Introduction

Tiger nut (*Cyperus esculentus* L.) is a type of cash crop with a high comprehensive utilization value. The underground tuber is rich in oil, starch, sugar, protein, dietary fibre and other nutrients [1–3]. The above-ground parts can be used as high-quality forage grass for livestock and poultry [4,5]. As a kind of multi-purpose crop native to desert areas, tiger nut has the characteristics of wide adaptability, high biological yield and high added value, so it has great potential for development and application [6]. However, the tubers need to be treated with warm water to break physical dormancy and improve sprouting. Yang *et al.* [7] indicated that sprouting was high when the tubers of *C. esculentus* were pre-soaked in warm water (35°C) compared with pre-soaking below 20°C. Moreover, even the size of the tubers from the same plant varied significantly, which might influence sprout establishment and later reproduction.

Dormancy is a common biological phenomenon in seeds, which is the temporary failure to complete germination under favourable conditions. In tiger nut the tuber coat is hard, which results in physical dormancy and reduces tuber viability [8]. However, even in the case of seeds, a hard coat is not really a case of dormancy in the strict sense. For example, many legume species possess seeds with extremely hard coats that delay germination for many years due to prevention of water uptake [5,9], but these seeds readily germinate once the coat is scarified [10]. Seed dormancy is one of the major factors affecting some species' germination rate and seedling emergence. For some species, seed dormancy significantly depends on temperature [11]. Brändel [12] found that temperatures between 3, 8 and 12°C were effective in relieving dormancy in *Bidens tripartita* seeds and the maximum germination was more than 80%. For *Acacia mearnsii*, the total germination and germination rate reached the highest values when the seeds were immersed in hot water at 80°C for 5 min before germination [13]. Cold stratification is also required to break epicotyl dormancy in the seeds of species such as *Paeonia ostia* [14].

Knowledge of seed biology is pivotal to understanding seedling establishment, succession and regeneration and the impact these processes have on plant communities [15]. Moreover, a seed is the reproductive body of a seed plant. The resource input from the seed to the offspring will directly affect the fitness of the offspring and thus affect the regeneration of vegetation communities [16]. It is widely assumed that seedlings from large seeds are more tolerant of environmental harshness because of their greater metabolic reserves and the production of larger and more robust seedlings compared with smaller seeds [17,18]. However, Baraloto *et al.* [19] reported that seedlings from small seeds of neotropical trees grew faster and overcame the initial size advantage associated with large seed size. Under different selection pressures, seeds of different sizes have different fates in their later life history. Seed vigor and early seedling growth are critical factors affecting crop production [20]. Nevertheless, Mourtzinis *et al.* [21] revealed that soybean (*Glycine max* L. Merr.) seed mass had no effect on seed yield during a two-year experiment.

Tiger nut is a crop of great potential globally, and the variation in its tuber size is substantial. Traditionally, larger seeds have greater advantage during the early stage of seedling development and the advantages would disappear after late growth and reproduction. Drawing parallels between true seed and the seed tubers of tiger nut, the objective of our investigation was to evaluate suitable treatment methods to release dormancy and accelerate the sprouting of various tuber sizes, and determine the response of different tuber sizes to sprout growth and later tuber reproduction.

## 2. Material and methods

### 2.1. Plant materials

Seed tubers of *C. esculentus* were harvested from farmland in Songyuan, Jilin province, China, in 2020 and the tubers were kept at 4°C to maintain viability until they were used. The tubers were selected according to three sizes: large (L, about 1.5 g), medium (M, about 0.8 g) and small (S, about 0.1 g).

### 2.2. Dormancy break

All tuber sizes were washed separately three times to remove soil before sprouting and five pre-sprouting treatments were used to break dormancy. A sprouting test was conducted on 30 seed-tuber samples placed on moist blotting paper in a growth cabinet at 35°C and exposed to 12 hours light daily for 11 days. The cold test was done according to the improved method of Elias and Copeland [22]. Different sizes of tubers were exposed to −2°C for 2 days on the moist blotting paper and then the tubers were sprouted as described above for 11 days. Three group sizes of tubers were immersed in warm water at either 25°C, 35°C or 45°C for 2 hours or maintained at room temperature (RT, mean 22°C, designated as the control treatment) before sprouting. Four replicates were used for each treatment and for each tuber size. Sprouted tubers were counted every day to determine the sprouting percentage and the sprouting rate. On the last day, tiller numbers were recorded and the sprouts were separated into shoots, roots and leftovers (plant parts with shoots and roots removed are designated as ‘leftovers’). The separated samples were put into individual paper bags and dried at 65°C for 3 days to determine shoot mass, root mass and sprout biomass allocation.

Sprouting rate = Σ(Si/Di), Si: number of normal sprouts on the ith day; Di: number of days until the ith reading [23];

Sprout biomass allocation = (shoot mass + root mass)/(shoot mass + root mass + leftover mass) × 100.

### 2.3. Later reproductive traits

Fifteen 20 cm diameter plastic pots were filled with farmland soil from the fields around Changchun City on 1 June 2021. Ten tubers of each size were sown in each pot after treatment with 35°C water for 2 hours. The sprouts were thinned to three individuals 10 days later and five replicates were used for each tuber size. All the pots were arranged in a randomized block design outdoors under a rain shelter at Jilin Jianzhu University (44°33 N, 123°82 31 E), which was made from transparent 200 µm polyethylene sheeting.

The plants were harvested on 1 September 2021 when all the above-ground parts were senescing. The roots and the soil were separated carefully to leave the tubers of each plant intact. The number of tillers per plant and the tuber number (the number of tubers produced by each plant) were recorded for all plants, and each plant was then separated into above-ground parts, tubers and leftover parts in the laboratory. Tubers from the separated plants were washed three times to remove soil. All the separated samples were put into individual paper bags and dried at 65°C for 3 days to a constant weight and then weighed separately to determine above-ground mass, tuber mass, tuber yield and tuber size.

Tuber mass: mass of all the tubers produced by a plant;

Tuber size: mass of tubers produced by a plant/number of the tubers produced by a plant.

### 2.4. Data analysis

All the datasets were initially tested for normal distribution using the Kolmogorov–Smirnov test and for variation homogeneity using Levene's test. All the data were subjected to analysis with one-way analysis of variance (ANOVA) using SPSS statistical software (version 17.0. SPSS Inc, Chicago, IL, USA). A two-way ANOVA was also used to determine the sprouting percentage, sprouting rate, tiller number, shoot mass, root mass, sprout biomass allocation and tuber yield under different pre-sprouting treatments over three tuber sizes. The least significant difference (LSD) test was used to determine the significant differences between treatments. The significance level was set at *p* = 0.05.

To determine the allometric relationships between plant mass and tuber mass, and tuber size and tuber number, above-ground mass, tuber mass, tuber size and tuber number were log transformed to fit a normal distribution. The classical ‘allometric’ model: log Y = a log X + b was used to analyse the allometric relationship via the log-transformed version, where a and b are the allometric slope and the log of the allometric coefficient, respectively. The slopes and intercepts were determined by standardized major axis (SMA) regression using the SMATR software package (standardized major axis tests & routines, [24]).

## 3. Results

### 3.1. Dormancy break and sprout growth

A two-way ANOVA indicated that both tuber size and treatment individually affected the sprouting percentage and sprouting rate, and similarly significant effects resulted from their interactions (table 1).

**Table 1.** Analysis of variance of sprouting percentage and sprouting rate among three different tuber sizes of *C. esculentus*. Note: *** denotes significant difference at *p* < 0.001.

	d.f.	F	*p*
sprouting			
size	4	28.622	<0.001***
treatment	2	86.929	<0.001***
size × treatment	8	12.384	<0.001***
sprouting rate			
size	4	26.785	<0.001***
treatment	2	56.841	<0.001***
size × treatment	8	6.867	<0.001***

**Figure 1.**
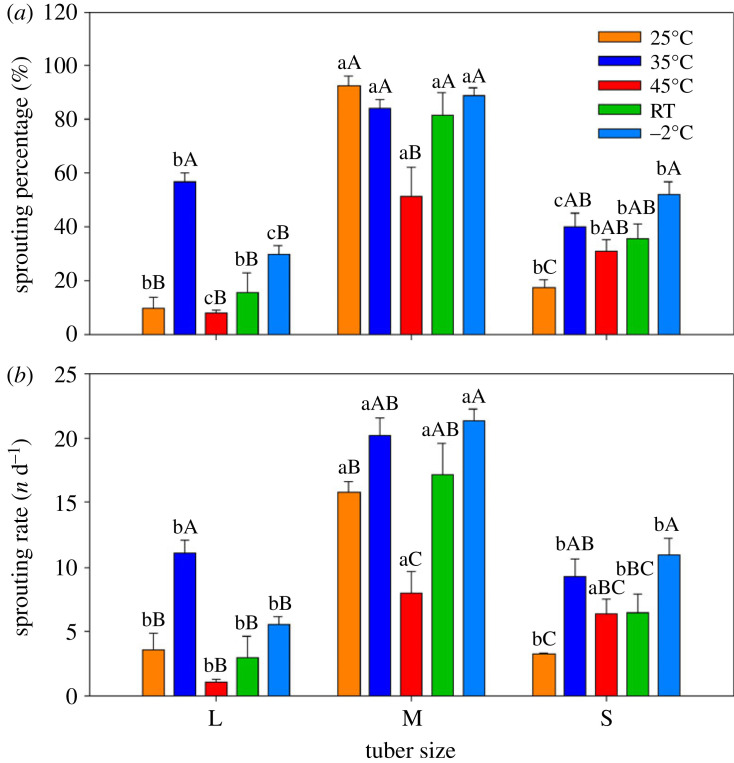
Bar graph showing (*a*) sprouting percentage (means ± 1 s.d.) and (*b*) sprouting rate (means ± 1 s.d.) among three tuber sizes of *C. esculentus*. Within the same treatment, different lower-case letters indicate a significant difference between tuber sizes at *p* < 0.05. Within the same tuber size, different capital letters indicate a significant difference between treatments at *p* < 0.05.

In particular, sprouting and sprouting rate varied significantly with tuber size under different pre-sprouting treatments (figure 1). Medium size tubers had higher sprouting percentages and sprouting rates than large and small tubers under all pre-sprouting treatments (figure 1*a*). The sprouting percentage in large tubers was higher than in small tubers following immersion in 35°C water for 2 hours before sprouting, while the sprouting percentage in small tubers was higher than in large tubers under the RT pre-sprouting treatment. The sprouting percentage in medium size tubers was similar under the 25°C, 35°C, RT and −2°C pre-sprouting treatments and markedly higher than under the 45°C treatment, and the highest sprouting values were more than 90%. A similar pattern was found in the sprouting rate (figure 1*b*).

A two-way ANOVA indicated that both tuber size and treatment individually affected tiller number, shoot mass, root mass and sprout biomass allocation, and similarly significant effects resulted from their interactions (table 2).

**Table 2.** Analysis of variance of tiller number, shoot mass, root mass and sprout biomass allocation among three tuber sizes of *C. esculentus*. Note: * denotes significant difference at *p* < 0.05; *** denotes significant difference at *p* < 0.001.

	d.f.	F	*p*-value
tiller number			
size	4	2.934	0.021*
treatment	2	7.641	0.001***
size × treatment	8	4.420	<0.001***
shoot mass			
size	4	27.524	<0.001***
treatment	2	24.890	<0.001***
size × treatment	8	12.449	<0.001***
root mass			
size	4	29.301	<0.001***
treatment	2	29.254	<0.001***
size × treatment	8	12.621	<0.001***
sprout biomass allocation			
size	4	42.821	<0.001***
treatment	2	23.586	<0.001***
size × treatment	8	6.302	<0.001***

**Figure 2.**
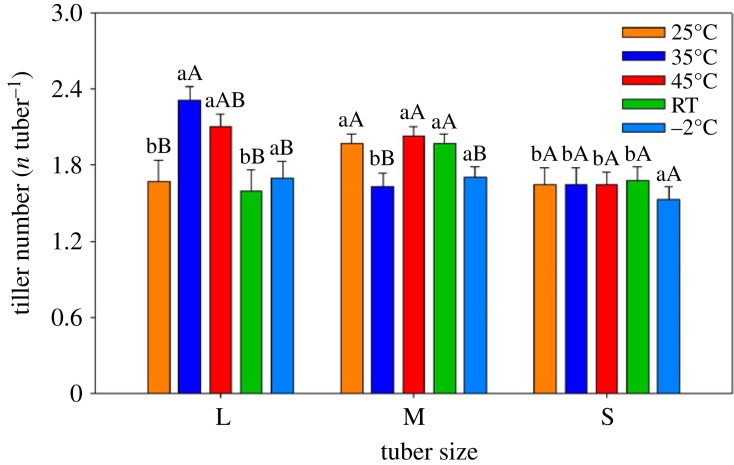
Bar graph showing tiller numbers (means ± 1 s.d.) among three tuber sizes of *C. esculentus*. Within the same treatment, different lower-case letters indicate a significant difference between tuber sizes at *p* < 0.05. Within the same tuber size, different capital letters indicate a significant difference between treatments at *p* < 0.05.

**Figure 3.**
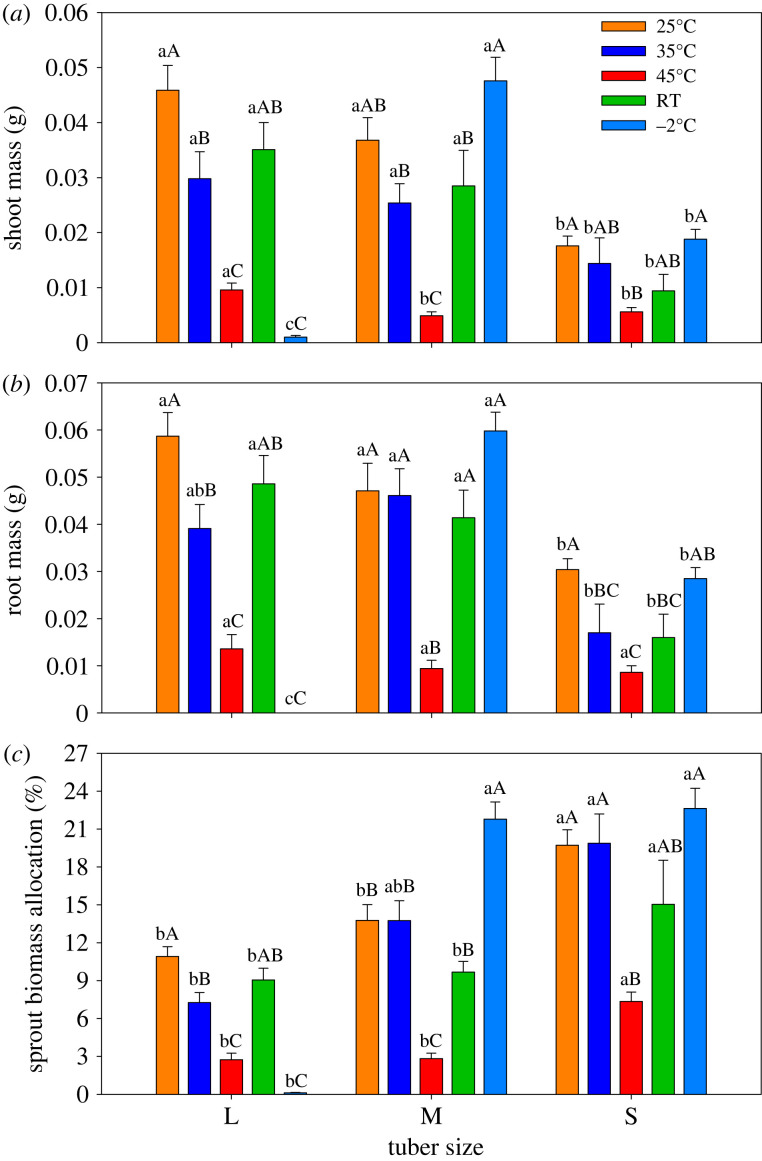
Bar graph showing (*a*) shoot mass (means ± 1 s.d.) (*b*) root mass (means ± 1 s.d.) and (*c*) sprout biomass allocation (means ± 1 s.d.) among three tuber sizes of *C. esculentus*. Within the same treatment, different lower-case letters indicate a significant difference between tuber sizes at *p* < 0.05. Within the same tuber size, different capital letters indicate a significant difference between treatments at *p* < 0.05.

Similar results were observed for tiller numbers between large and medium size tubers when the tubers were treated with 45°C hot water, whereas tiller numbers decreased markedly in small tubers except for under −2°C treatments. However, tuber size did not affect tiller number under the −2°C treatment. The maximum values for tiller numbers in large tubers were observed under the 35°C and 45°C pre-sprouting treatments. Tiller numbers in medium size tubers reached minimum values under the 35°C and −2°C treatments. Tiller numbers in small tubers were similar under all treatments (figure 2). Significant variations were found in shoot mass and root mass among different tuber sizes within treatments (figure 3). Shoot mass from large and medium size tubers was similar and significantly higher than from small tubers under 25°C, 35°C and the RT treatments. Shoot mass reached its maximum values when large tubers were treated with the 35°C water treatment. In contrast, the highest shoot mass was found when small tubers were cultured under the −2°C treatment (figure 3*a*). The patterns of root mass were similar to those of shoot mass (figure 3*b*). The sprout biomass allocation was higher in small tubers than medium and large tubers under the RT, 25°C, 35°C and 45°C treatments. Sprout biomass allocation in medium and small tubers remained steady and was significantly higher than in large tubers under the −2°C treatment (figure 3*c*).

### 3.2. Later reproduction traits

**Figure 4.**
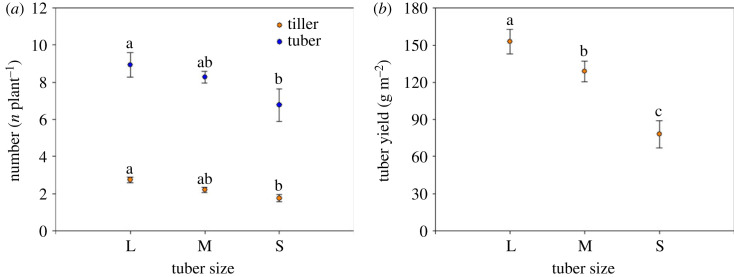
Bar graph showing (*a*) tiller and tuber number (means ± 1 s.d.) and (*b*) tuber yield (means ± 1 s.d.) among three tuber sizes of *C. esculentus* after harvesting. Within the same treatment, different lower-case letters indicate a significant difference between tuber sizes at *p* < 0.05.

**Figure 5.**
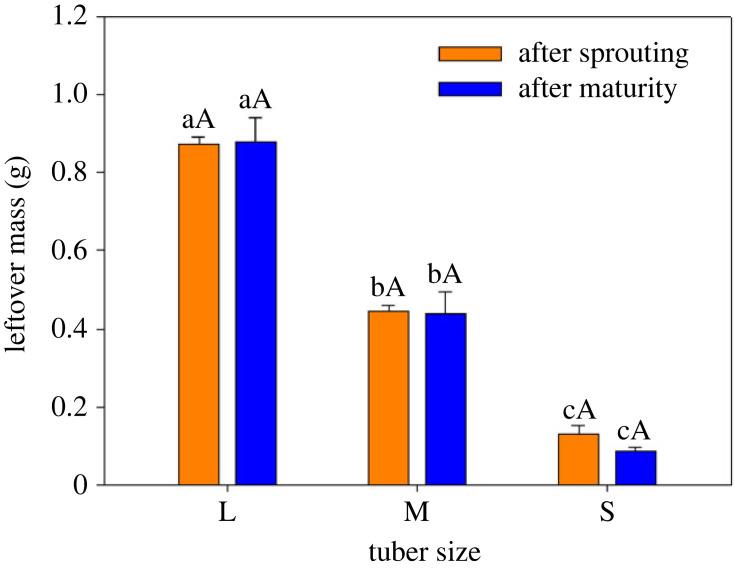
Bar graph showing leftover mass after sprouting and maturity (means ± 1 s.d.) among three tuber sizes of *C. esculentus*. Within the same treatment, different lowercase letters indicate a significant difference between tuber sizes at *p* < 0.05. Within the same tuber size, different capital letters indicate a significant difference between treatments at *p* < 0.05.

The results for tiller and tuber number were very similar and varied among the seed tuber size groups (figure 4). The large tubers had significantly higher tiller numbers and tuber yields than small tubers, with the tiller numbers and yields of the former being 2.7 per plant and 153.0 g m^−2^, respectively. Tiller numbers and tuber yields from medium tubers were not significantly different from either the large or the small tubers (figure 4*a*). Tuber yield decreased sharply with a decrease in tuber size, and the mass of tubers harvested from the large tubers was twice as heavy as the small tubers (figure 4*b*). The change in the patterns of leftover mass from different seed tuber sizes after sprouting and maturity were very similar (figure 5). Leftover mass after sprouting and maturity decreased markedly with a decrease in the seed tuber size. Large tubers had the greatest maximum values for leftover mass after sprouting and maturity, with values of 0.8733 and 0.8786 g, respectively. Small tubers had the lowest leftover mass after sprouting and maturity, the values being 0.1293 and 0.0857 g, respectively. Within the seed tuber size groups there were no significant differences found between leftover mass after sprouting and maturity (figure 5).

**Figure 6.**
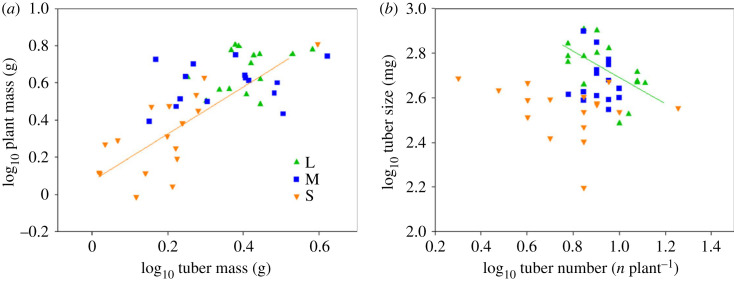
Allometric relationships between plant mass and tuber mass (*a*), and tuber size and tuber number (*b*) of *C. esculentus*.

No allometric relationship between plant mass versus tuber mass was observed, except in plant mass from small tubers (figure 6*a* and table 3). The slope of the relationship between plant mass and tuber size in small tubers was significantly greater than 1 (the lower and upper 95% confidence intervals were 1.128 and 2.309 for small tubers, respectively; table 3). By contrast, significant negative correlation between tuber size and tuber number was found in large tubers (figure 6*b*; *p* = 0.027, table 3).

**Table 3.** Effects of tuber size on the slopes and intercepts of allometric relationships between log (plant mass) and log (tuber mass), log (tuber size) and log (tuber number) of *C. esculentus*, using the standardized major axis on the allometric model log_10_Y = slope × log_10_X + intercept. Note: * denotes significant difference at *p* < 0.05; *** denotes significant difference at *p* < 0.001.

treatment	L	M	S
log (tuber mass) versus log (plant mass)			
slope	1.390	0.821	1.614
intercept	0.104	0.304	0.006
*R*^2^	0.146	0.059	0.558
CI of slope	0.819–2.358	0.473–1.427	1.128–2.309
*p*	0.159	0.385	0.001***
log (tuber size) versus log (tuber number)			
slope	−1.005	−1.613	−0.545
intercept	3.673	4.153	2.966
*R*^2^	0.322	0.014	0.072
CI of slope	−1.617–0.625	−2.836−0.917	−0.906–0.328
*p*	0.027*	0.671	0.297

## 4. Discussion

### 4.1. Dormancy break and sprout growth

The present results clearly demonstrated that medium-sized tubers performed better than small and large tubers during sprouting (figure 1). It is known in seeds that the effects of seed size on germination vary among species. Gadisa [25] indicated that small to medium-sized seeds have better rates of germination and seedling vigour than bigger seeds. Other investigations have shown that the germination rate increased with increasing seed weight because of the greater metabolic reserves of large seeds [16,26]. Sprout growth from large and medium tubers was better than from small tubers in our investigation (figure 3), paralleling findings in seedlings, which have low photosynthetic rates and therefore depend mostly on the amount of reserve nutrients that are stored in the seed for their initial growth [27]. Large variation in seed size is an important trait that may have consequences for growth and survival of seedlings, and in our case, the performance of tiger nut sprouts. In general, seedling size is positively correlated with seed size and mass [28,29].

The 35°C, −2°C and RT treatments increased sprouting in medium tubers of *C. esculentus*, while higher temperature treatment decreased sprouting and sprout growth markedly. To be released from physical dormancy, the seeds of some species must experience certain environmental factors to improve germination [30,31]. Lastuvka *et al.* [32] confirmed that dormancy release occurred following the storage of seed at 2 or 5°C during stratification. Surprisingly, tubers not subjected to any pre-sprouting treatment (RT) demonstrated better sprouting and sprout development in our study. To be released from dormancy, an external stimulus is necessary to overcome the seed dormancy of some species for germination to occur under natural conditions [33–35].

### 4.2. Later reproduction traits

The seed source influences plant growth and yield during the growing season. For example, plant growth and grain yield increased with an increase in the seed size of bread wheat (*Tritium aestivum* L.) [25]. In *C. esculentus*, tiller number and yield decreased as the tuber size decreased. Further, a plant's morphology, e.g. the number of meristems that can develop into reproductive tiller branches, is one of the important factors influencing seed yield [36]. The yields from wheat cultivars with small seeds (2.0–2.2 mm) have been reported as being significantly lower than yields from large seeds (greater than 2.2–2.5 mm) [37]. Along similar lines, the current work indicated that the highest tuber yields were obtained from medium and large seed tuber sizes, whereas the lowest tuber yields were obtained from small seed tuber sizes. Some of the impacts of small seeds (and small tuber sizes) may be a lack nutrients and production of weak plants that are very sensitive to diseases, and have high mortality and low yield [38]. It is obvious that seed size effects on seedlings persist into adulthood and that reproduction and seed size for some species is an important physical indicator of performance and yield [39].

Under the reserve effect hypothesis, larger seeds have more stored resources than smaller ones. The results of our study were consistent with this part of the hypothesis. Further, in hazardous environments most of the resources not committed during seedling development should be held in reserve to provision the shoot and root after sprouting and seedling establishment [40,41]. Nevertheless, there was no difference in leftover mass within seed tuber size groups between sprouting and maturity (figure 5). This indicated that the sprouts did not consume any uncommitted resources stored in their tubers until maturity. The reason for this might be that the sprouts were able to obtain enough resources through their shoots and roots to satisfy their growth. Moreover, the tubers being difficult to rot and mould in the soil for high dry matter ratio, rich in oil and starch, the leftover could be harvested together with plant.

## 5. Conclusion

Great variation in tuber size was one of the key factors influencing the breaking of dormancy, sprout establishment and later tuber yield in *C. esculentus*. The results presented here demonstrate that temperature treatments of 25°C, 35°C and −2°C for 2 hours pre-sprouting improved sprouting and subsequent sprout growth. An important finding is that tubers not subjected to any pre-sprouting treatment (RT) responded and behaved as well as the treated one (certainly for medium tubers) during sprouting and seedling development in our study. Moreover, sprouting and sprout growth in medium tubers was better than in large and small tubers under all pre-sprouting treatments. Our findings also showed that tuber number and yield increased with increasing tuber size. Because *C. esculentus* can be used as cash crop with high utilization value, information about the need for relieving physical dormancy and improving sprouting will be helpful in its production. For this reason, we recommend that medium size tubers be selected as seed tubers and sowed directly for their competitive advantage in production. Seed quality and the stress resistance of various size tubers should be focused on in the future, which is important in production.

## Data Availability

The file about our data was provided as the electronic supplementary material. Data deposited in Dryad Digital Repository: https://doi.org/10.5061/dryad.76fk344 [42].
